# Presumptive brain influx of large neutral amino acids and the effect of phenylalanine supplementation in patients with Tyrosinemia type 1

**DOI:** 10.1371/journal.pone.0185342

**Published:** 2017-09-26

**Authors:** Willem G. van Ginkel, Danique van Vliet, Johannes G. M. Burgerhof, Pim de Blaauw, M. Estela Rubio Gozalbo, M. Rebecca Heiner-Fokkema, Francjan J. van Spronsen

**Affiliations:** 1 Department of Metabolic Diseases, Beatrix Children’s Hospital, University Medical Center Groningen, University of Groningen, Groningen, The Netherlands; 2 Department of Epidemiology, University Medical Center Groningen, University of Groningen, Groningen The Netherlands; 3 Department of Laboratory Medicine, University Medical Center Groningen, University of Groningen, Groningen, The Netherlands; 4 Department of Pediatrics and Laboratory Genetic Metabolic Diseases, Maastricht University Medical Center, Maastricht, The Netherlands; Nathan S Kline Institute, UNITED STATES

## Abstract

**Introduction:**

Hereditary Tyrosinemia type 1 (HT1) is a rare metabolic disease caused by a defect in the tyrosine degradation pathway. Current treatment consists of 2-(2-nitro-4-trifluoromethylbenoyl)-1,3-cyclohexanedione (NTBC) and a tyrosine and phenylalanine restricted diet. Recently, neuropsychological deficits have been seen in HT1 patients. These deficits are possibly associated with low blood phenylalanine concentrations and/or high blood tyrosine concentrations. Therefore, the aim of the present study was threefold. Firstly, we aimed to calculate how the plasma amino acid profile in HT1 patients may influence the presumptive brain influx of all large neutral amino acids (LNAA). Secondly, we aimed to investigate the effect of phenylalanine supplementation on presumptive brain phenylalanine and tyrosine influx. Thirdly, we aimed to theoretically determine minimal target plasma phenylalanine concentrations in HT1 patient to ensure adequate presumptive brain phenylalanine influx.

**Methods:**

Data of plasma LNAA concentrations were obtained. In total, 239 samples of 9 HT1 children, treated with NTBC, diet, and partly with phenylalanine supplementation were collected together with 596 samples of independent control children. Presumptive brain influx of all LNAA was calculated, using Michaelis-Menten parameters (K_m_) and V_max_-values obtained from earlier articles.

**Results:**

In HT1 patients, plasma concentrations and presumptive brain influx of tyrosine were higher. However, plasma and especially brain influx of phenylalanine were lower in HT1 patients. Phenylalanine supplementation did not only tend to increase plasma phenylalanine concentrations, but also presumptive brain phenylalanine influx, despite increased plasma tyrosine concentrations. However, to ensure sufficient brain phenylalanine influx in HT1 patients, minimal plasma phenylalanine concentrations may need to be higher than considered thus far.

**Conclusion:**

This study clearly suggests a role for disturbed brain LNAA biochemistry, which is not well reflected by plasma LNAA concentrations. This could play a role in the pathophysiology of the neuropsychological impairments in HT1 patients and may have therapeutic implications.

## Introduction

Hereditary Tyrosinemia type I (HT1; McKusick 276700) is an inborn error of metabolism caused by a deficiency in the catabolic pathway of tyrosine. Due to a genetic defect in the enzyme fumarylacetoacetate hydrolase (FAH), several toxic products accumulate, causing liver failure, renal tubulopathy, rickets, cardiomyopathy, porphyria like syndrome, and hepatocellular carcinoma [1]. Since 1992, HT1 patients are treated with 2-(2-nitro-4-trifluoromethylbenoyl)-1,3-cyclohexanedione (NTBC) [2]. NTBC inhibits tyrosine catabolism upstream from the primary enzymatic defect (at the level of 4-OH-phenylpyruvate dioxygenase), preventing the formation of the toxic products, and thereby substantially improving clinical outcome [3]. NTBC treatment strongly increases plasma tyrosine concentrations, necessitating dietary restriction of tyrosine and its precursor phenylalanine [1,4]. Such combined treatment of NTBC and diet may still result in high plasma tyrosine concentrations, while phenylalanine concentrations are often low [5–8]. While previous studies have shown the possible importance of phenylalanine supplementation in HT1 patients, the minimum plasma phenylalanine target level remains to be established [5,8].

In the past few years, neurocognitive impairments have been observed in HT1 patients [6,9–13]. The pathophysiological mechanisms underlying these neurocognitive impairments are not fully understood. The metabolic derangement in HT1 with high plasma tyrosine and low phenylalanine concentrations is supposed to play a central role. However, the resulting transport of these amino acids across the blood-brain barrier (BBB) could be even more important, as this could directly influence brain amino acid and neurotransmitter concentrations [8,14].

At the BBB, all large neutral amino acids (LNAA) are primarily transported by the so-called L-type amino acid transporter 1 (LAT1) in a competitive manner [15–17]. The transport rate of each individual LNAA is determined by its plasma concentration, and the K_m_-value (the plasma concentration at which the transport rate is 50% of its maximum), which differs for each LNAA [18,19]. Under physiological conditions, LAT1 is saturated for >95% by these LNAA [19]. Therefore, high plasma concentrations of a particular LNAA would not only increase brain influx of this LNAA but also outcompete brain uptake of the other LNAA [18–20].

The competitive transport mechanism has for example been shown to exist in phenylketonuria (PKU) mice [21] and maple syrup urine disease (MSUD) mice [22]. In these mice, Vogel et al. (2014) clearly showed that plasma amino acid concentrations in PKU and MSUD mice represent only a partial picture of brain amino acid homeostasis. Next to this, therapeutically, the addition of a specific amino acid that interferes with the transport of the other amino acids at the BBB has shown to improve the outcome in some defects of amino acid metabolism such as guanidinoacetate methyltransferase deficiency, pyridoxine dependent epilepsy and MSUD [23–25]. Therefore, taking together these theoretical considerations, experimental, and clinical data, the competitive transport of amino acids across the BBB shows to be important in various inborn errors of amino acid metabolism [26].

Therefore, the aim of the present study was threefold. Firstly, we aimed to calculate how the plasma amino acid profile as observed in HT1 patients on NTBC and dietary treatment may influence the presumptive brain influx of all LNAA compared to healthy controls. Secondly, we aimed to investigate the effect of phenylalanine supplementation on presumptive brain phenylalanine and tyrosine influx in four of these HT1 patients. Thirdly, we aimed to theoretically determine minimal plasma phenylalanine concentrations in HT1 patients to ensure adequate presumptive brain phenylalanine influx.

## Methods

### Patients

In total, 239 plasma samples of nine HT1 patients (six males, three females) were obtained between 2002 and 2015, from diagnosis or first measurement after introduction of NTBC and diet (median: 0.53 years; range: 0.04–12.2 years) till the current age with a maximum of 18 years (median: 11.5 years; range: 2.8–18.0 years). Six patients were diagnosed primarily suffering from liver failure, among others characterized by severe coagulopathy due to decreased synthetic liver function. For these patients, data from the first period after diagnosis were excluded until coagulopathy had been restored (defined by prothrombin time <16 sec and activated partial prothrombin time <35 sec) to avoid samples with high methionine concentrations associated with liver failure. Three other patients were diagnosed due to neonatal screening or because of an affected sibling. For these patients, only the first measurement after diagnosis was excluded for adjustment to the NTBC and dietary treatment, if liver synthesis function was still normal. All patients were treated with NTBC (1–2 mg/kg/day) and a tyrosine and phenylalanine restricted diet in the University Medical Center Groningen or Maastricht University Medical Center. Four of these patients were treated with phenylalanine supplementation ranging from 10 to 32 mg/kg/day next to their regular NTBC and dietary treatment. Of all 239 plasma samples, 73 were obtained during phenylalanine supplementation.

In total, 596 anonymized amino acid profiles from independent control participants (mean: 4.8 years, all younger than 18 years old) were obtained. Control participants were patients who underwent analysis of plasma amino acid concentrations as part of the diagnostic process in the hospital, but who eventually did not have an inborn error of amino acid metabolism.

All HT1 patients and/or their parents or guardians gave written informed consent for retrospective analysis of their data, acknowledging that one patient had died and no informed consent was obtained. This study was approved by the Medical Ethical Committee of the University Medical Center Groningen.

### Biochemical analyses

Plasma samples of both HT1 patients and control participants had been taken in the clinic, not taking into account the timing of plasma sampling and its relation to the last meal of the patient or control individuals. In these samples, concentrations of LNAA (except for tryptophan) and glutamine had been quantified in deproteinized plasma by cation-exchange high-performance liquid chromatography followed by post-column ninhydrin derivatization, using norleucine as an internal standard, on a Biochrom 20 or 30 analyser (Pharmacia Biotech, Cambridge, UK).

### Calculations of presumptive brain amino acid influx

Plasma LNAA concentrations were used to calculate presumptive brain influx of individual LNAA. Brain influxes were calculated using classical Michaelis-Menten parameters (K_m_) and V_max_-values for each LNAA as reported by Smith et al. 2000, measured with an in situ rat brain perfusion technique [19]. For each LNAA, substrate inhibition (K_app_) was calculated in the presence of competing amino acids using previously published equations [24].

$$K_{app=K_{m}\;[1+\sum_{1\to n}{\left(C_{i}/K_{i}\right)}_{n}]}$$

In this equation, K_m_ (μM) is the substrate concentration at which the reaction rate is half of the V_max_. C_i_ is the current plasma concentration of each competitive amino acid (μM), and K_i_ is the K_m_ of these respective inhibiting amino acids (μM). The resulting K_app_ shows the current inhibitory effect of all LNAA together. This value is used to determine the presumptive brain influx using the following equation: brain influx = (V_max_)*(C)/(K_app_+C). In this equation, brain influx is the presumptive brain uptake of the LNAA (nanomoles per minute per gram of brain tissue (nmol/(min*g))), V_max_ is the maximum transport velocity (nmol/(min*g)), and C is the plasma concentration of the amino acid (μM).

In all NTBC and dietary treated HT1 patients, Z-scores for both plasma concentrations and presumptive brain influxes of individual LNAA were determined. Z-scores were calculated by subtracting the mean value of the control participants from the values of the patients, and dividing this difference by the standard deviation of the control values. Afterwards mean Z-scores for individual patients were calculated.

To assess the effect of phenylalanine supplementation on presumptive brain phenylalanine and tyrosine influx, we investigated the four HT1 patients with the lowest plasma phenylalanine concentrations who were subsequently treated without and with phenylalanine supplementation in addition to the regular NTBC and dietary treatment. Plasma phenylalanine and tyrosine concentrations, plasma phenylalanine/tyrosine (phe/tyr) ratios, and presumptive brain phenylalanine and tyrosine influx in four HT1 patients before and during phenylalanine supplementation were calculated.

Thereafter, for all HT1 patients, plasma phenylalanine concentrations that would probably result in sufficient brain phenylalanine influx (determined as mean, -1, and -2 standard deviation (SD) below the mean values of presumptive brain influx in control participants) were calculated, if all LNAA concentrations in the HT1 patients would remain the same. To do so, the previously used equation for brain influx was rewritten into: C = (brain influx*K_app_)/(V_max_-brain influx). To estimate these minimum target plasma phenylalanine concentrations for HT1 patients, the mean brain influx as well as the -1 SD and -2 SD of the mean brain influx of control participants were used in the equation, together with the previously calculated individual K_app_-values_._

### Statistics

The distribution of brain influx of LNAA was compared between HT1 patients and controls using multiple Mann Whitney U tests. Considering the fact that we have repeated observations within each patient and independent data of 596 controls, we used the following testing procedure. To test the null hypothesis that the observations of patients and controls originated from equal distributions, we repeatedly took one random observation from each patient and all observations from the controls. We calculated for each (sub)dataset the p-value, using the Mann-Whitney U test. After 1000 tests for each LNAA, we evaluated the distributions of p-values. To evaluate the effect of phenylalanine supplementation in HT1 patients, means of plasma values and presumptive brain influx of phenylalanine and tyrosine before and after supplementation were calculated for each patient. Correlational analyses were performed in each individual patient to assess whether age was correlated with presumptive brain phenylalanine influx in HT1 patients. In control participants, linear regression analysis was done after logarithmic transformation to assess a possible correlation between age and presumptive brain phenylalanine influx. Linear mixed effect models with variance components for random effects were used to investigate a possible correlation between presumptive brain phenylalanine influx and plasma tyrosine concentrations, plasma phenylalanine concentrations, and phe/tyr ratios in HT1 patients without receiving phenylalanine supplementation. In all statistical tests, a (mean) p-value of <0.05 was considered statistically significant. Analyses were conducted with the statistical program SPSS 22 (IBM, Chicago, Illinois).

## Results

### Plasma concentrations and presumptive brain influx of individual LNAA in HT1 patients and controls

Plasma LNAA concentrations without phenylalanine supplementation were repetitively measured in all patients, ranging from 1 to 40 times. Table 1 shows mean plasma concentrations of all LNAA (except for tryptophan) and glutamine for HT1 patients and control participants calculated from the mean concentrations of each HT1 individual. Plasma phenylalanine concentrations were significantly lower (in 99% out of 1000 tests *p*<0.05; mean *p* = 0.00462), while plasma tyrosine concentrations were much higher (in 100% out of 1000 tests *p*<0.05; mean *p*<0.001) in HT1 patients compared to control participants. Plasma concentrations of all other LNAA were not significantly different between HT1 patients and controls.

**Table 1:** Plasma amino acid concentrations.

	HT1	Controls	Mean *P*-value
Phenylalanine	27 ±14	54 ± 16	0.00462
	33 [16–38]	50 [43–61]	
Tyrosine	384 ± 53	64 ± 23	2.67·10^−7^
	382 [337–432]	60 [49–74]	
Valine	226 ± 26	188 ± 54	0.199
	230 [208–246]	181 [155–213]	
Isoleucine	59 ± 8	55 ± 22	0.494
	59 [52–64]	52 [41–64]	
Leucine	115 ± 18	105 ± 39	0.454
	116 [101–129]	99 [81–121]	
Methionine	23 ± 6	21 ± 9	0.401
	21 [18–27]	19 [15–25]	
Histidine	81 ± 11	83 ± 22	0.572
	79 [73–89]	81 [70–94]	
Threonine	129 ± 37	118 ± 62	0.427
	114 [109–160]	106 [84–134]	
Glutamine	480 ± 46	517 ± 121	0.382
	488 [446–507]	513 [451–578]	

Plasma amino acid concentrations (μmol/l) in HT1 patients treated with NTBC and diet and controls. Data are presented as Mean ± SD and Median with IQR.

**Fig 1:**
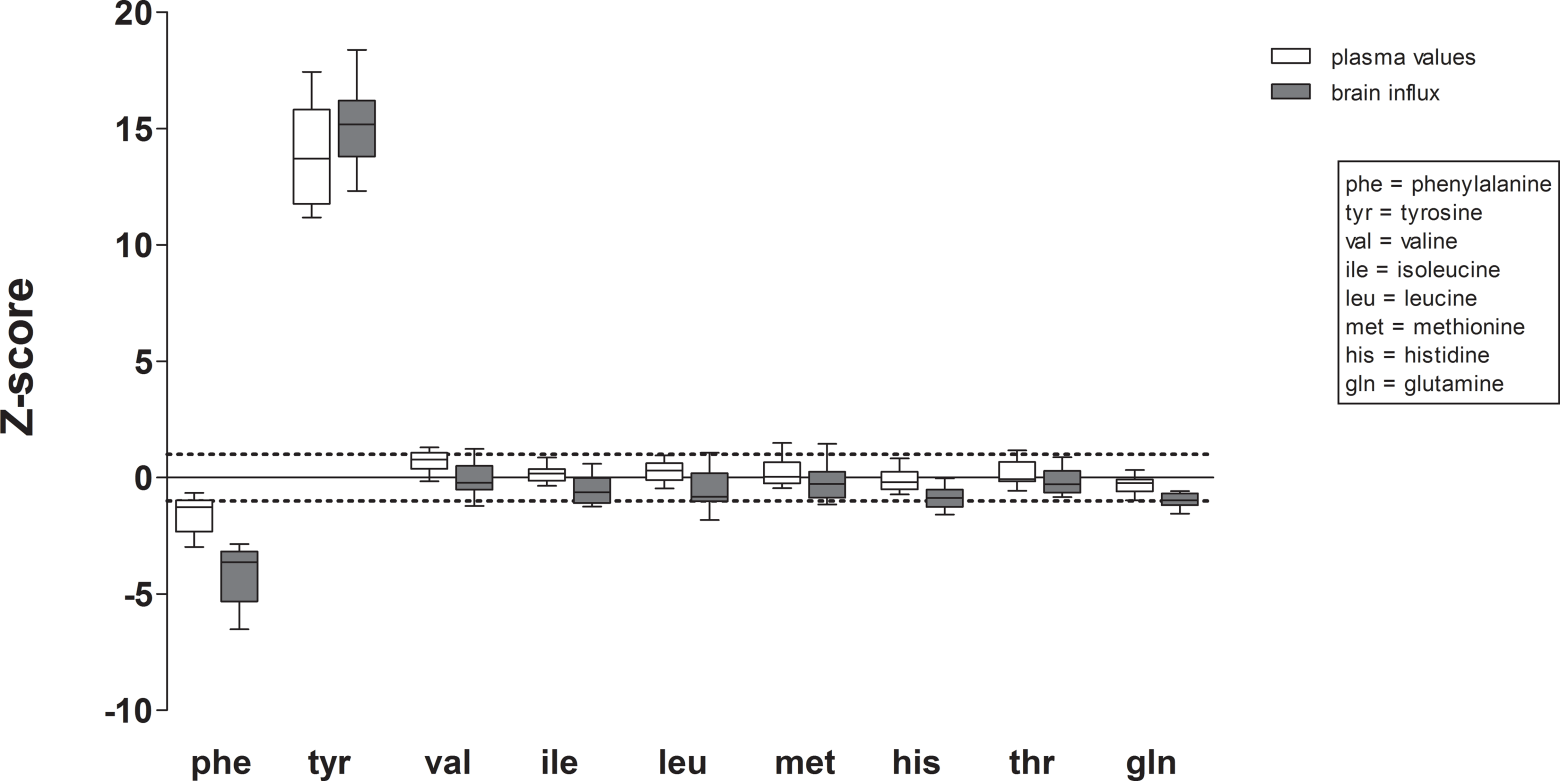
Plasma concentrations and presumptive brain influx of individual LNAA in HT1 patients. Mean plasma concentrations and presumptive brain influx with SD of individual LNAA in HT1 patients expressed as Z-scores (individual patient means) of values in control participants. Dashed lines represent Z-scores of -1 and +1.

Fig 1 shows the mean plasma concentrations and presumptive brain influx of the measured LNAA and glutamine in HT1 patients not receiving phenylalanine supplementation, expressed as Z-scores of values in control participants. Mean plasma tyrosine concentrations and presumptive brain tyrosine influx in HT1 patients were higher than values in control participants with Z-scores of 13.8 and 15.2, respectively. In contrast, mean plasma phenylalanine concentrations in HT1 patients tended to be slightly lower than in controls with a Z-score of -1.6, while mean presumptive brain phenylalanine influx was much lower with a Z-score of -4.2. Mean plasma concentrations and presumptive brain influxes of other LNAA were all between Z-scores of -0.9 and 0.7.

### Effect of phenylalanine supplementation on presumptive brain phenylalanine and tyrosine influx in HT1 patients

Table 2 shows the biochemical effects of phenylalanine supplementation in four HT1 patients. Descriptive analyses show that, on phenylalanine supplementation, mean plasma phenylalanine and tyrosine concentrations increased in these four patients. The proportional increase of plasma phenylalanine concentrations was higher than the increase of plasma tyrosine concentrations in 2 patients, resulting in higher phe/tyr ratios in these patients. On phenylalanine supplementation, the increase in plasma phenylalanine concentrations tended to be accompanied by an increase of the mean presumptive brain influx of phenylalanine in 3 patients. Although plasma tyrosine concentrations increased on phenylalanine supplementation in all patients, presumptive brain influx of tyrosine only increased in two of these patients.

**Table 2:** The effect of phenylalanine supplementation.

	Patient 1		Patient 2		Patient 3		Patient 4	
**Number of samples**	37	20	40	15	6	21	1	17
**Phe supplementation (mg/kg/day)**	No	Yes15-20	No	Yes 10–20	No	Yes11-23	No	Yes 15–32
**Plasma phenylalanine (μmol/L)**	23	34	23	26	8	16	5	19
**Plasma tyrosine (μmol/L)**	401	491	342	360	336	426	324	396
**Plasma phe/tyr ratio**	0.065	0.066	0.074	0.073	0.024	0.036	0.015	0.048
Brain phenylalanine influx (nmol/(min*g)	4.9	6.2	5.5	5.4	1.9	2.8	1.1	3.9
Brain tyrosine influx (nmol/(min*g)	32.8	37.6	31.5	29.7	30.1	29.4	28.7	33.2

Results on plasma biochemistry and presumptive brain phenylalanine and tyrosine influx in four HT1 patients before and during phenylalanine supplementation, expressed as means.

### Presumptive minimal target plasma phenylalanine concentrations in HT1 patients

**Fig 2:**
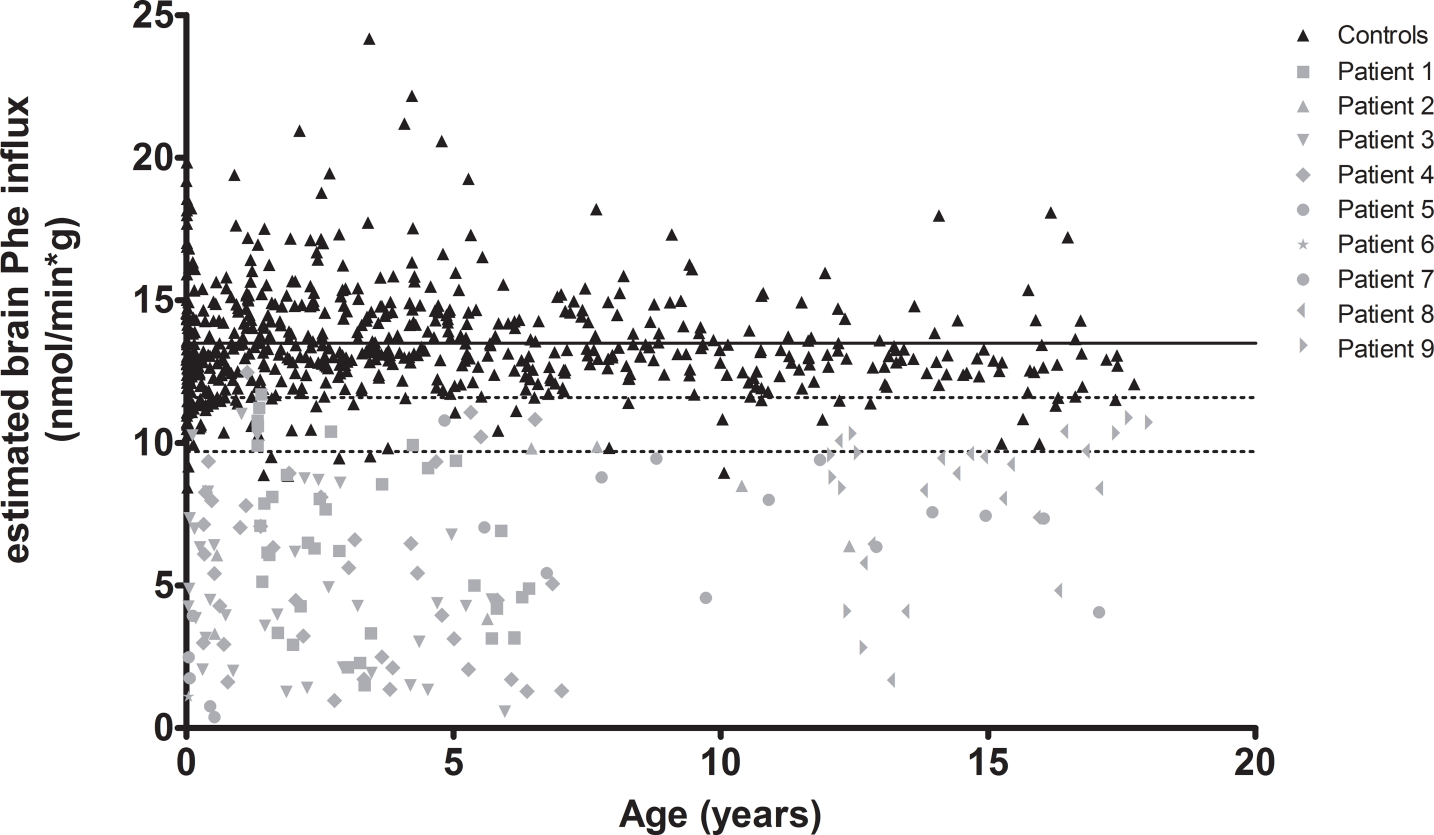
Presumptive brain influx of phenylalanine comparing HT1 patients and controls. Presumptive brain influx of phenylalanine of both HT1 patients and controls plotted against age. The straight line represents the mean presumptive brain phenylalanine influx of controls. The dashed lines represent the -1SD and -2SD of the presumptive brain influx of controls.

In Fig 2, presumptive brain phenylalanine influx is plotted against age for both HT1 patients and controls. Presumptive brain phenylalanine influx in HT1 patients was significantly lower than in controls (in 100% out of 1000 tests *p*<0.05; mean *p*<0.001). In individual HT1 patients, no clear effect of age on the presumptive brain phenylalanine influx could be seen, although age was significantly correlated with presumptive brain phenylalanine influx in two (out of nine) patients (*r* = -0.448; *p* = 0.006 and *r* = 0.562; *p* = 0.001). However, the correlation coefficient was opposite in both patients. In control participants, age was not significantly correlated with presumptive brain phenylalanine influx (*p* = 0.100).

Linear mixed effect model analyses on presumptive brain phenylalanine influx and parameters of plasma amino acid (tyrosine and phenylalanine concentrations and phe/tyr ratios) biochemistry revealed that presumptive brain phenylalanine influx was most strongly correlated with plasma phenylalanine concentrations (*p*<0.001, *β* = 0.167, *t* = 23.57). Correlations between presumptive brain phenylalanine influx and plasma tyrosine concentrations and plasma phe/tyr ratios were less strong based on Akaike’s Information Criteria.

Given the strong correlations between presumptive brain phenylalanine influx and plasma phenylalanine concentrations, minimal plasma phenylalanine concentrations were calculated to ensure sufficient presumptive brain phenylalanine influx in HT1 patients. Mean presumptive brain phenylalanine influx in HT1 patients not receiving phenylalanine supplementation was calculated to be 5.5 ± 2.5 nmol/(min*g) and 13.5 ± 1.9 nmol/(min*g) (-1SD: 11.6; -2SD: 9.7 nmol/(min*g)) in control participants. To reach mean, -1SD, or -2SD values of the presumptive brain phenylalanine influx of control participants, plasma phenylalanine concentrations in HT1 patients needed to be increased from 27 to 84 ± 5, 68 ± 4, and 53 ± 3 μmol/L, respectively, if plasma concentrations of all other amino acid concentrations would remain the same.

## Discussion

This study investigated (1) plasma LNAA concentrations and presumptive brain influx of individual LNAA in HT1 patients in comparison to control participants, (2) the effect of phenylalanine supplementation in HT1 patients on plasma LNAA biochemistry as well as on presumptive brain LNAA influx, and (3) minimal plasma phenylalanine concentrations necessary to increase the presumptive brain phenylalanine influx. The main findings of this study are threefold. Firstly, although plasma phenylalanine concentrations were low to normal, the presumptive brain influx of phenylalanine was largely impaired in HT1 patients compared to control participants. Secondly, phenylalanine supplementation tended to improve the presumptive brain phenylalanine influx in HT1 patients, having only a small negative effect on plasma tyrosine concentrations and presumptive brain tyrosine influx. Thirdly, to ensure sufficient brain phenylalanine influx in HT1 patients, minimal plasma phenylalanine concentrations may need to be higher than considered thus far.

Before discussing these results in more detail, some methodological issues are addressed. With regard to the plasma amino acid measurements, unfortunately, plasma tryptophan concentrations could not be studied and therefore possible disturbances in presumptive brain tryptophan influx with implications for cerebral serotonin metabolism could not be investigated. In addition, the timing of blood sampling and fasting status is unfortunately unknown. It is known that there is a large variation of plasma phenylalanine concentrations in HT1 patients during the day, with low concentrations especially occurring in the afternoon [5,7]. With regard to calculations for presumed brain influx, the K_m_ and V_max_-values used in this study have been determined in rats. However, although absolute values differ between humans and rats, LNAA transport characteristics, as determined in brain microvascular endothelial cells of humans, have shown a strong correlation to those in rats [27]. Thus, we assume that inter-species differences are not a major issue. We acknowledge that the presumptive brain influx of LNAA, based on LNAA transport characteristics and plasma concentrations, do not directly reflect brain LNAA availability. However, as lumbar puncture to obtain cerebral spinal fluid is rather invasive and brain phenylalanine concentrations could not be measured by normal magnetic resonance spectroscopy techniques, this theoretical method is used. In addition, the calculated presumptive brain amino acid influx has already been shown useful in the design of arginine-fortified and LNAA-optimized amino acid supplements in glutaric aciduria type 1 and MSUD [24,28].

Our results clearly suggest a non-optimal brain LNAA homeostasis in HT1 patients. With regard to our first research question, except for tyrosine and phenylalanine, no strong differences in plasma LNAA concentrations between HT1 patients and controls were observed. Plasma tyrosine concentrations were largely increased, although still within the recommended range for HT1 patients [1]. Plasma phenylalanine concentrations were decreased as also previously reported by various study groups [5,7,8]. All other LNAA were within the normal range. Presumptive brain influx of individual LNAA, however, showed a different profile. While presumptive brain tyrosine influx was similarly increased in HT1 patients compared to controls as were plasma tyrosine concentrations, this was not true for other LNAA, especially phenylalanine. Presumptive brain phenylalanine influx in HT1 patients compared to controls was much more decreased than could be expected only based on plasma phenylalanine values. Moreover, presumptive brain influx of all other LNAA was slightly reduced in HT1 patients compared to controls (although mostly within the normal range with z-scores around -1), despite normal plasma concentrations. Although the clinical significance of this disturbed brain LNAA biochemistry requires further investigation, the observed neurocognitive impairments in HT1 patients suggest a possible relationship.

Regarding the second research question, previous studies have shown dietary interventions in inborn errors of amino acid metabolism to be very effective in increasing brain LNAA concentrations and thereby possibly improving the neurocognitive outcome [22,25,26]. Phenylalanine supplementation in HT1 patients has not only been related to increased plasma phenylalanine concentrations and disappearance of eczema as a peripheral consequence of low plasma phenylalanine concentrations, but has also been suggested to be associated with improved brain functioning with better psychomotor development and disappearance of cortical myoclonus [8]. In the present study, plasma phenylalanine as well as tyrosine concentrations seem to increase after phenylalanine supplementation. Despite higher plasma tyrosine concentrations and resulting competition for transport across the BBB, presumptive brain phenylalanine influx was increased, although it should be noticed that values of control participants are never reached. Only one patient (patient 2, Table 2) seemed not to respond to the phenylalanine supplementation. Plasma phenylalanine nor tyrosine concentrations were increased in this patient, possibly caused by an amount of supplementation that was too low or problems with dietary compliance.

With regard to the third research question, based on the results of the present study, plasma phenylalanine concentrations in HT1 patients would need to be increased more than considered thus far to ensure adequate brain phenylalanine influx. In previous studies in HT1 patients, hypophenylalaninemia was defined by plasma phenylalanine concentrations below 40 μmol/L [7] or below 30 μmol/L [8]. Based on our study (without the inclusion of tryptophan), plasma phenylalanine concentrations need to be increased even further to maintain adequate presumptive brain influx of phenylalanine in HT1 patients. Unfortunately, this estimated brain influx is based on a theoretical model not knowing real brain phenylalanine concentrations in HT1 patients. Thimm et al. showed increased tyrosine concentrations and decreased concentrations of serotonin metabolites in the cerebral spinal fluid in HT1 patients, but did not report on concentrations of other LNAA such as phenylalanine [14]. However, low concentrations of serotonin metabolites could indicate that tryptophan, the precursor of serotonin, is outcompeted by tyrosine at the BBB, like we suggest with this model for phenylalanine.

## Conclusion

The pathophysiological mechanisms of neuropsychological impairment in HT1 are not fully understood and need further investigation. This theoretical experiment suggests a role for disturbed brain LNAA transport. While plasma tyrosine concentrations and presumptive brain tyrosine influx were similarly markedly elevated in HT1 patients, presumptive brain phenylalanine influx was much more affected than could be expected only based on its plasma values. To improve presumptive brain phenylalanine influx in HT1 patients, additional plasma phenylalanine supplementation has shown to be effective, but it seems that plasma phenylalanine concentrations need to be increased even further than was previously suggested.

## Abbreviations

BBB: blood-brain barrier
HT1: Hereditary Tyrosinemia type 1
LNAA: Large Neutral Amino Acid
LAT1: L-type amino acid transporter 1
MSUD: Maple Syrup Urine Disease
NTBC: 2-(2-nitro-4-trifluoromethylbenoyl)-1,3-cyclohexanedione
phe/tyr ratio: phenylalanine/tyrosine ratio
PKU: Phenylketonuria

## References

[1] de Laet C, Dionisi-Vici C, Leonard JV, McKiernan P, Mitchell G, Monti L, et al Recommendations for the management of tyrosinaemia type 1. Orphanet J Rare Dis 2013 1 11;8:8-1172-8-8. 10.1186/1750-1172-8-8 PMC3558375 23311542

[2] Lindstedt S, Holme E, Lock EA, Hjalmarson O, Strandvik B. Treatment of hereditary tyrosinaemia type I by inhibition of 4-hydroxyphenylpyruvate dioxygenase. Lancet 1992 10 3;340(8823):813–817. 1383656 10.1016/0140-6736(92)92685-9

[3] Holme E, Lindstedt S. Tyrosinaemia type I and NTBC (2-(2-nitro-4-trifluoromethylbenzoyl)-1,3-cyclohexanedione). J Inherit Metab Dis 1998 8;21(5):507–517. 9728331 10.1023/a:1005410820201

[4] Russo PA, Mitchell GA, Tanguay RM. Tyrosinemia: a review. Pediatr Dev Pathol 2001 May-Jun;4(3):212–221. 11370259 10.1007/s100240010146

[5] Wilson CJ, Van Wyk KG, Leonard JV, Clayton PT. Phenylalanine supplementation improves the phenylalanine profile in tyrosinaemia. J Inherit Metab Dis 2000 11;23(7):677–683. 11117429 10.1023/a:1005666426079

[6] De Laet C, Munoz VT, Jaeken J, Francois B, Carton D, Sokal EM, et al Neuropsychological outcome of NTBC-treated patients with tyrosinaemia type 1. Dev Med Child Neurol 2011 10;53(10):962–964. doi: 10.1111/j.1469-8749.2011.04048.x 21745202 10.1111/j.1469-8749.2011.04048.x

[7] Daly A, Gokmen-Ozel H, MacDonald A, Preece MA, Davies P, Chakrapani A, et al Diurnal variation of phenylalanine concentrations in tyrosinaemia type 1: should we be concerned? J Hum Nutr Diet 2012 4;25(2):111–116. doi: 10.1111/j.1365-277X.2011.01215.x 22168396 10.1111/j.1365-277X.2011.01215.x

[8] van Vliet D, van Dam E, van Rijn M, Derks TG, Venema-Liefaard G, Hitzert MM, et al Infants with Tyrosinemia Type 1: Should phenylalanine be supplemented? JIMD Rep 2014 9 26. 10.1007/8904_2014_358 PMC4361924 25256450

[9] Masurel-Paulet A, Poggi-Bach J, Rolland MO, Bernard O, Guffon N, Dobbelaere D, et al NTBC treatment in tyrosinaemia type I: long-term outcome in French patients. J Inherit Metab Dis 2008 2;31(1):81–87. doi: 10.1007/s10545-008-0793-1 18214711 10.1007/s10545-008-0793-1

[10] Pohorecka M, Biernacka M, Jakubowska-Winecka A, Biernacki M, Kusmierska K, Kowalik A, et al Behavioral and intellectual functioning in patients with tyrosinemia type I. Pediatr Endocrinol Diabetes Metab 2012;18(3):96–100. 23146787

[11] Thimm E, Richter-Werkle R, Kamp G, Molke B, Herebian D, Klee D, et al Neurocognitive outcome in patients with hypertyrosinemia type I after long-term treatment with NTBC. J Inherit Metab Dis 2012 3;35(2):263–268. doi: 10.1007/s10545-011-9394-5 22069142 10.1007/s10545-011-9394-5

[12] Bendadi F, de Koning TJ, Visser G, Prinsen HC, de Sain MG, Verhoeven-Duif N, et al Impaired cognitive functioning in patients with tyrosinemia type I receiving nitisinone. J Pediatr 2014 2;164(2):398–401. doi: 10.1016/j.jpeds.2013.10.001 24238861 10.1016/j.jpeds.2013.10.001

[13] van Ginkel WG, Jahja R, Huijbregts SC, Daly A, MacDonald A, De Laet C, et al Neurocognitive outcome in tyrosinemia type 1 patients compared to healthy controls. Orphanet J Rare Dis 2016 6 29;11(1):87-016-0472-5. 10.1186/s13023-016-0472-5 PMC4928338 27356512

[14] Thimm E, Herebian D, Assmann B, Klee D, Mayatepek E, Spiekerkoetter U. Increase of CSF tyrosine and impaired serotonin turnover in tyrosinemia type I. Mol Genet Metab 2011 2;102(2):122–125. doi: 10.1016/j.ymgme.2010.11.003 21112803 10.1016/j.ymgme.2010.11.003

[15] del Amo EM, Urtti A, Yliperttula M. Pharmacokinetic role of L-type amino acid transporters LAT1 and LAT2. Eur J Pharm Sci 2008 10 2;35(3):161–174. doi: 10.1016/j.ejps.2008.06.015 18656534 10.1016/j.ejps.2008.06.015

[16] Killian DM, Chikhale PJ. Predominant functional activity of the large, neutral amino acid transporter (LAT1) isoform at the cerebrovasculature. Neurosci Lett 2001 6 22;306(1–2):1–4. 11403943 10.1016/s0304-3940(01)01810-9

[17] Smith QR, Momma S, Aoyagi M, Rapoport SI. Kinetics of neutral amino acid transport across the blood-brain barrier. J Neurochem 1987 11;49(5):1651–1658. 3668544 10.1111/j.1471-4159.1987.tb01039.x

[18] Pardridge WM. Blood-brain barrier carrier-mediated transport and brain metabolism of amino acids. Neurochem Res 1998 5;23(5):635–644. 9566601 10.1023/a:1022482604276

[19] Smith QR. Transport of glutamate and other amino acids at the blood-brain barrier. J Nutr 2000 4;130(4S Suppl):1016S–22S. 10736373 10.1093/jn/130.4.1016S

[20] Fernstrom JD, Wurtman RJ. Brain serotonin content: physiological regulation by plasma neutral amino acids. Science 1972 10 27;178(4059):414–416. 5077329 10.1126/science.178.4059.414

[21] van Vliet D, Bruinenberg VM, Mazzola PN, van Faassen MH, de Blaauw P, Kema IP, et al Large Neutral Amino Acid Supplementation Exerts Its Effect through Three Synergistic Mechanisms: Proof of Principle in Phenylketonuria Mice. PLoS One 2015 12 1;10(12):e0143833 doi: 10.1371/journal.pone.0143833 26624009 10.1371/journal.pone.0143833 PMC4666635

[22] Vogel KR, Arning E, Wasek BL, McPherson S, Bottiglieri T, Gibson KM. Brain-blood amino acid correlates following protein restriction in murine maple syrup urine disease. Orphanet J Rare Dis 2014 5 8;9:73-1172-9-73. 10.1186/1750-1172-9-73 PMC4022424 24886632

[23] Schulze A, Ebinger F, Rating D, Mayatepek E. Improving treatment of guanidinoacetate methyltransferase deficiency: reduction of guanidinoacetic acid in body fluids by arginine restriction and ornithine supplementation. Mol Genet Metab 2001 12;74(4):413–419. doi: 10.1006/mgme.2001.3257 11749046 10.1006/mgme.2001.3257

[24] Strauss KA, Wardley B, Robinson D, Hendrickson C, Rider NL, Puffenberger EG, et al Classical maple syrup urine disease and brain development: principles of management and formula design. Mol Genet Metab 2010 4;99(4):333–345. doi: 10.1016/j.ymgme.2009.12.007 20061171 10.1016/j.ymgme.2009.12.007 PMC3671925

[25] Coughlin CR,2nd, van Karnebeek CD, Al-Hertani W, Shuen AY, Jaggumantri S, Jack RM, et al Triple therapy with pyridoxine, arginine supplementation and dietary lysine restriction in pyridoxine-dependent epilepsy: Neurodevelopmental outcome. Mol Genet Metab 2015 Sep-Oct;116(1–2):35–43. doi: 10.1016/j.ymgme.2015.05.011 26026794 10.1016/j.ymgme.2015.05.011

[26] van Vliet D, Derks TG, van Rijn M, de Groot MJ, MacDonald A, Heiner-Fokkema MR, et al Single amino acid supplementation in aminoacidopathies: a systematic review. Orphanet J Rare Dis 2014 1 13;9:7-1172-9-7. 10.1186/1750-1172-9-7 PMC3895659 24422943

[27] Hargreaves KM, Pardridge WM. Neutral amino acid transport at the human blood-brain barrier. J Biol Chem 1988 12 25;263(36):19392–19397. 2848825

[28] Strauss KA, Brumbaugh J, Duffy A, Wardley B, Robinson D, Hendrickson C, et al Safety, efficacy and physiological actions of a lysine-free, arginine-rich formula to treat glutaryl-CoA dehydrogenase deficiency: focus on cerebral amino acid influx. Mol Genet Metab 2011 Sep-Oct;104(1–2):93–106. doi: 10.1016/j.ymgme.2011.07.003 21820344 10.1016/j.ymgme.2011.07.003

